# The complete chloroplast genome of *medinilla arboricola* (melastomataceae) and its phylogenetic analysis

**DOI:** 10.1080/23802359.2026.2749904

**Published:** 2026-10-20

**Authors:** Hongxin Wang, Langxing Yuan, Yongxing He, Yiran Liu

**Affiliations:** ^a^ Zhai Mingguo Academician Work Station, University of Sanya, Sanya, Hainan, China; ^b^ Tropical Crops Genetic Resources Institute, Chinese Academy of Tropical Agricultural Sciences, Haikou, Hainan, China; ^c^ Sanya Nanfan Research Institute, Hainan University, Haikou, Hainan, China

**Keywords:** Organellar DNA, genomic resource, phylogenomics, illumina sequencing

## Abstract

*Medinilla arboricola* is a near threatened climbing shrub endemic to Hainan Island, China. We present its first complete chloroplast genome, assembled using Illumina paired-end sequencing. The 155,517 bp plastome has a typical quadripartite structure: an 85,379 bp large single-copy region, a 16,568 bp small single-copy region, and two 26,785 bp inverted repeats, with a GC content of 37.02%. A total of 128 annotated genes were identified, comprising 84 protein-coding, 36 tRNA, and eight rRNA genes. Phylogenetic analyses based on 14 Melastomataceae plastomes placed *Medinilla arboricol*a among the sampled *Medinilla* species, as sister to *Medinilla tapete*-*magicum* with maximum support. This genome provides valuable plastomic data for taxonomic and evolutionary studies of *Medinilla* and Melastomataceae.

## Introduction

With approximately 6,000 described species, Melastomataceae is one of the world’s most diverse angiosperm families, distributed mainly across tropical and subtropical regions (Ulloa Ulloa et al. 2022). Tribe Sonerileae is a major ecologically diverse lineage, and its largest genus, *Medinilla* Gaudich. ex DC., is the third most species-rich genus in Melastomataceae A. L. Jussieu (Liu et al. 2022, 2024; Penneys et al. 2022; Quakenbush et al. 2025). *Medinilla* comprises shrubs, climbers and epiphytes, defined by isomorphic stamens, reduced connectives and three anther appendages (two ventral, one dorsal) (Regalado 1995), yet extensive morphological variation complicates its taxonomic circumscription (Chen and Renner 2007; Zhou et al. 2022; Chen et al. 2023).

Chloroplast genomes are small (typically 120–160 kb), structurally conserved and mostly uniparentally inherited, and have been widely used to resolve phylogenetic relationships, to infer maternal parentage and phylogeographic patterns, and to develop molecular markers such as simple sequence repeats (Dong et al. 2025; Wu et al. 2026). Such plastome resources are therefore valuable for both evolutionary studies and conservation applications.

*Medinilla arboricola* F. C. How is a climbing shrub endemic to Hainan Island, China (Figure 1), occurring in shaded lower‑to‑middle elevation forests (Chen and Renner 2007). It is listed as Near Threatened (NT) in the China Biodiversity Red List—Higher Plants (2020) (Ministry of Ecology and Environment of the People’s Republic of China, & Chinese Academy of Sciences, 2023). Several *Medinilla* species, including *M. arboricola*, are promising woody ornamentals (Wu et al. 2020). However, genomic data are needed to delimit species within this still poorly resolved genus (Khakhlova and Bock 2006; Kartonegoro et al. 2021; Penneys et al. 2022).

**Figure 1.**
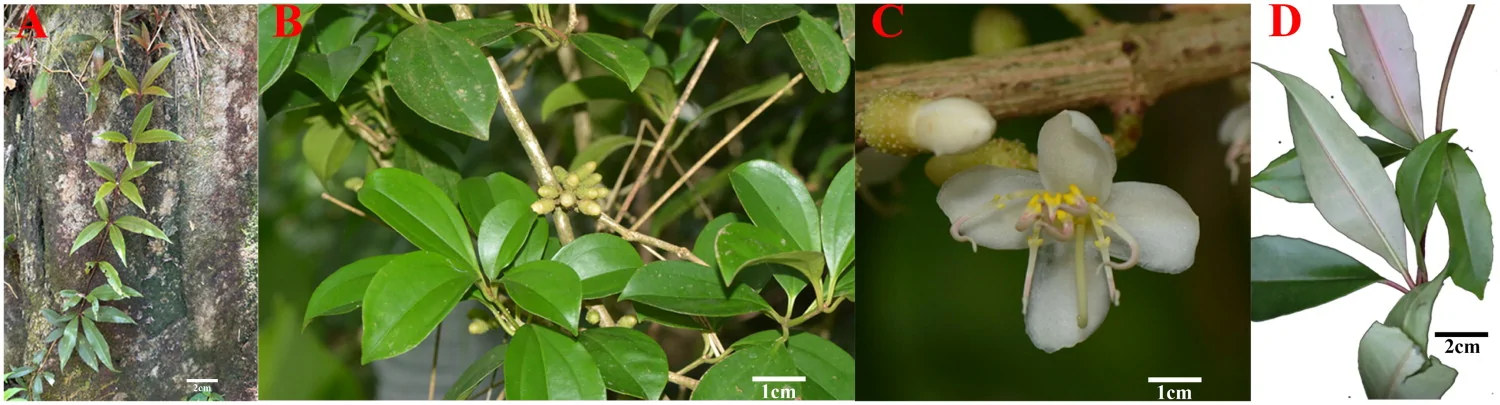
Plant image of *Medinilla arboricola* F. C. How. (A) Plants of *Medinilla arboricola* climbing on rocks. (B) Leaves and stems of *Medinilla arboricola*. Stems grayish yellow, quadrangular, glabrous. Leaves in whorls of 3–5, leaf blades coriaceous or subcoriaceous, elliptic or ovate-elliptic. (C) Flowers of *Medinilla arboricola*. Petals white, oblong, 2–3 cm long. Stamens 8–10, 12–15 mm long; anthers linear-lanceolate, falcate, unequal in length. (D) Abaxial surface of leaves of *Medinilla arboricola*. Basal veins prominent, lateral veins indistinct. Photos were taken by Hongxin Wang in sanya, Hainan Province.

In this study, the complete chloroplast genome of *M. arboricola* was assembled, annotated and compared with related Melastomataceae plastomes. The results enrich the plastome resources of *Medinilla* and provide a reference for future comparative genomics, phylogenetic and population genetic studies of *Medinilla* and Melastomataceae.

## Materials and methods

Fresh leaves of one individual were collected from Maogan Village, Maogan Township, Baoting Li Autonomous County, Hainan Province, China (18.60529°N, 109.51416°E, 558 m). A specimen was deposited at Herbarium of the Institute of Tropical Crop Germplasm Resources, Chinese Academy of Tropical Agricultural Sciences (https://www.ctcgris.cn/en, contact: Langxing Yuan, orange269@163.com) under the voucher number YMD20241004.

Total genomic DNA was extracted using a Rapid Plant Genomic DNA Isolation Kit (Sangon Biotech). Short-insert (350 bp) libraries were prepared from the fragmented total genomic DNA following the manufacturer’s protocol and sequenced on the BGISEQ500 platform (BGI, China), yielding 7.7 Gb of raw data (150 bp paired-end reads). Raw reads were quality-controlled with fastp v1.3.1 and used for the de novo assembly of the complete chloroplast genome in GetOrganelle v1.5.2a with default settings (Jin et al. 2020). The assembly was verified by mapping clean reads back to it to confirm even, gap-free coverage (Figures S1 and S2) and by visualizing the assembly graph in Bandage v0.8.1 (Wick et al. 2015). Genome annotations were completed using a combination of GeSeq (Tillich et al. 2017) and CPGAVAS2 (Shi et al. 2019), with auxiliary tRNA boundary validation using tRNAscan. A circular genome map was generated using OGDRAW v1.3.1 (Greiner et al. 2019), and cis- and trans-splicing genes were identified using CPGView (Liu et al. 2023). The complete chloroplast genome was submitted to GenBank under accession number PZ746659.

**Figure 2.**
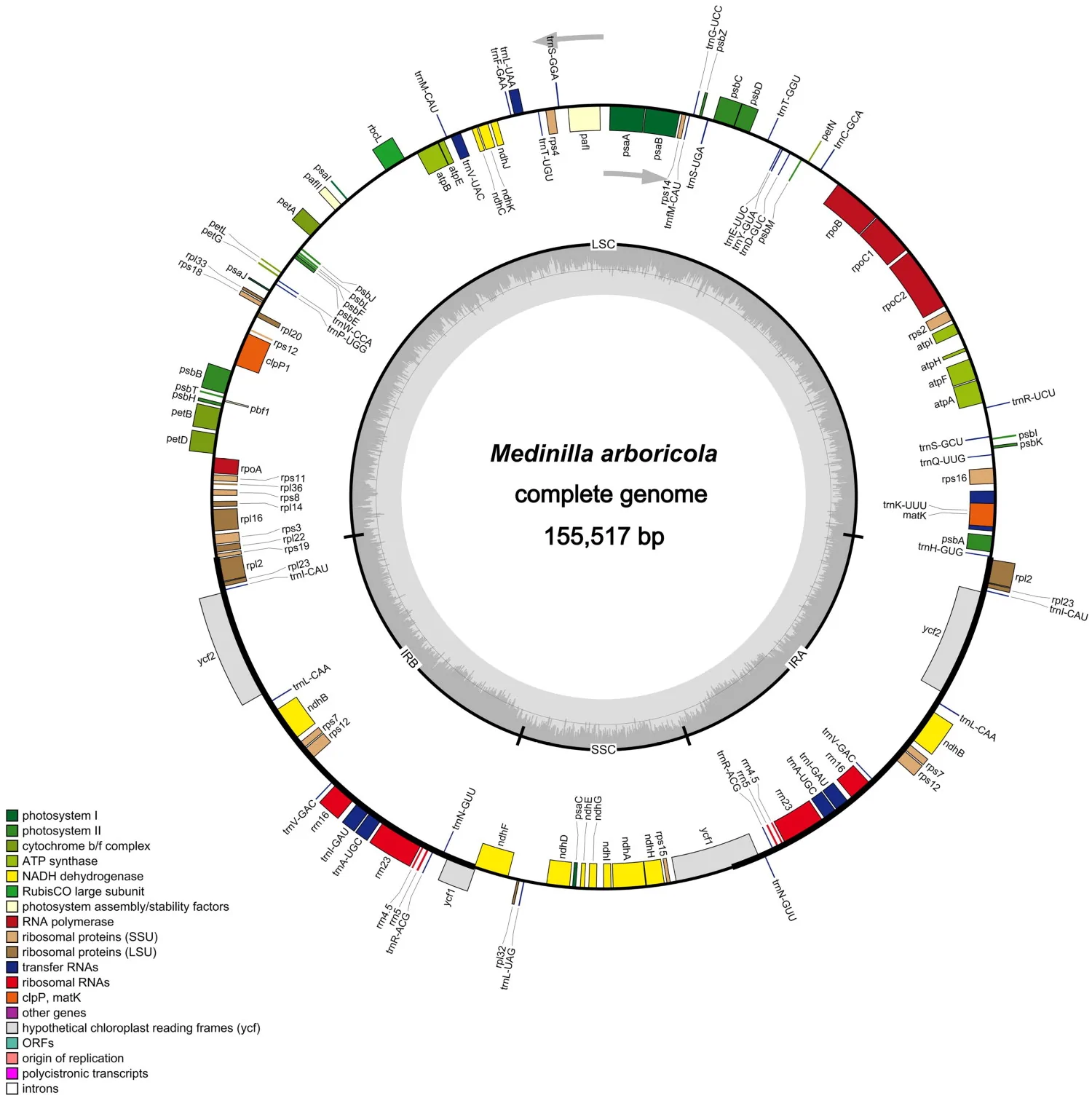
The circular chloroplast genome map of *medinilla arboricola*. The gray arrows showed the transcriptional direction of genes. Different colors represent genes with other functions. The inner circle’s gray portion indicates the chloroplast genome’s GC content.

To infer the phylogenetic position of *M. arboricola*, 14 species of Melastomataceae with available chloroplast genomes were selected, including two species from the genus *Dissochaeta* Blume (1831) as outgroups. Whole chloroplast genome sequences were aligned using MAFFT v7 with the –auto mode (Katoh and Standley 2013), and ambiguously aligned regions were trimmed using trimAl v1.5 with the –automated1 option (Capella-Gutiérrez et al. 2009). Maximum-likelihood (ML) phylogenetic analysis was conducted in IQ-TREE 2 (Minh et al. 2020), with the best-fitting nucleotide substitution model (TVM+F + R3) selected *via* ModelFinder (Kalyaanamoorthy et al. 2017) under the Bayesian Information Criterion (BIC), using 1,000 bootstrap replicates. Bayesian inference (BI) was performed in MrBayes v3.2.7 under the GTR+F + I + G4 model, running two million Markov chain Monte Carlo generations, discarding the first 25% of trees as burn-in, and confirming stationarity with effective sample sizes (ESS) > 200. Both ML and BI trees were visualized in FigTree v1.4.4 (http://tree.bio.ed.ac.uk/software/figtree/).

## Results

The chloroplast genome is 155,517 bp and has a typical quadripartite structure (Figure 2), consisting of a large single copy (LSC) region (85,379 bp), a small single-copy (SSC) region (16,568 bp), and two inverted repeat (IR) regions (26,785 bp each). The GC content was 37.02%. Quality control and read coverage are shown in Figures S1 and S2. The total lengths of 14 Melastomataceae chloroplast genomes ranged from 155,073 bp (MK814186) to 156,790 bp (MK994850) (Table S1).

A total of 128 genes were annotated, comprising 84 protein-coding genes (PCGs), 36 transfer RNA genes (tRNAs) and eight ribosomal RNA genes (rRNAs). Excluding the copies duplicated in the inverted repeat (IR) regions, the plastome contains 111 unique genes (78 PCGs, 29 tRNAs and four rRNAs). The IR-duplicated copies comprise six PCGs (*ndhB, rpl2, rpl23, rps7, rps12* and *ycf2*), seven tRNAs (*trnN-GUU*, *trnR-ACG*, *trnA-UGC*, *trnI-GAU*, *trnV-GAC*, *trnL-CAA* and *trnI-CAU*), and four rRNAs (*rrn5*, *rrn4.5*, *rrn23* and *rrn16*). Nine PCGs contain a single intron (*rps16, atpF*, *rpoC1*, *petB*, *petD*, *rpl16*, *rpl2*, *ndhB* and *ndhA*), and two PCGs (*pafI* and *clpP1*) contain two introns (Figure S3). Five tRNA genes (*trnK-UUU*, trnL-UAA, *trnV-UAC*, *trnI-GAU* (2×)*, and trnA-UGC* (2×)) contain a single intron. The rps12 gene is trans-spliced: its 5′ exon is located in the LSC, whereas its two 3′ exons are duplicated in the IRs (Figure S4).

ML and BI phylogenetic trees yielded consistent topologies (Figure 3). *M. arboricola* was placed within the sampled *Medinilla* species as sister to *Medinilla tapete*-*magicum* Cámara-Leret & Veldkamp, with maximum support (ML bootstrap support (BS) = 100%; Bayesian posterior probability (PP) = 1.00).

**Figure 3.**
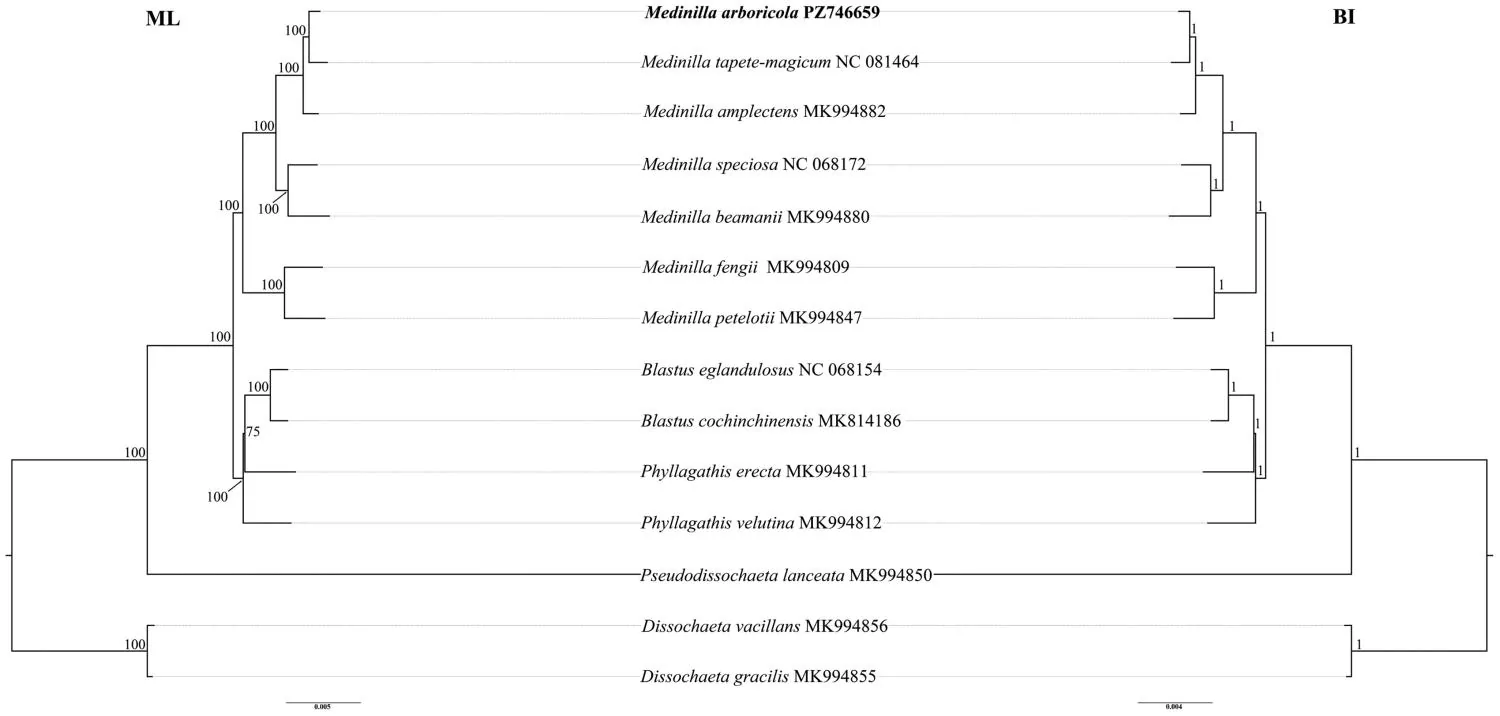
Maximum likelihood (left) and Bayesian (right) phylogenetic tree inferred from 14 chloroplast genomes using the whole length, *Dissochaeta vacillans* and *dissochaeta gracilis* were used as the outgroups. The sample with black bold font was the chloroplast genome of *Medinilla arboricola*. The following sequences were used: *Blastus eglandulosus* NC_068154 (Zhou et al. 2022), *Blastus cochinchinensis* MK814186 (Wen et al. 2019), *Phyllagathis erecta* MK994811 (Priyadi et al. 2024), *phyllagathis velutina* MK994812 (Priyadi et al. 2024), *medinilla fengii* MK994809 (Priyadi et al. 2024), *medinilla petelotii* MK994847 (Priyadi et al. 2024), *medinilla amplectens* MK994882 (Priyadi et al. 2024), *medinilla beamanii* MK994880 (Priyadi et al. 2024), *medinilla speciosa* NC_068172 (Zhou et al. 2022), *medinilla arboricola* PZ746659 (this study), *medinilla tapete magicum* NC_081464 (Zhou et al. 2022), *pseudodissochaeta lanceata* MK994850 (Priyadi et al. 2024), *dissochaeta vacillans* MK994856 (Priyadi et al. 2024) and *dissochaeta gracilis* MK994855 (Priyadi et al. 2024).

## Discussion and conclusion

Angiosperm chloroplast genomes are structurally and compositionally conserved, typically 120–160 kb with 110–130 unique genes (Cauz-Santos 2025). In line with this pattern, the genome of *M. arboricola* shows a typical quadripartite structure and a gene repertoire similar to other *Medinilla* genomes (Priyadi et al. 2024). Among sampled congeners, genome lengths range from 155,103 to 155,841 bp, with differences reflecting IR-boundary contraction/expansion and no gene loss or rearrangement consistent with the structural conservatism of Melastomataceae (Priyadi et al. 2024; Cauz-Santos 2025). The plastome phylogeny places *M. arboricola* as sister to *M. tapete-magicum* (BS = 100%; PP = 1.00; Figure 3), within a strongly supported *Medinilla* clade, consistent with its morphological traits (Chen and Renner 2007). Recent phylogenomic studies clarified the expanded monophyly of *Medinilla*, which now incorporates the formerly independent genera *Pachycentria* Blume and *Plethiandra* Hook.f. (Quakenbush et al. 2025).

The close relationship between *M. arboricola* and Southeast Asian congeners suggests affinities between the Hainan and Malesian *Medinilla* floras, but this inference, based on plastome data alone, should be regarded as preliminary. Denser sampling of Hainan endemics, nuclear loci and molecular dating analyses are needed to test the origin and diversification of *Medinilla* on Hainan Island (Liu et al. 2024; Priyadi et al. 2024; Quakenbush et al. 2025).

In conclusion, we sequenced, assembled and characterized the complete chloroplast genome of the near threatened Hainan endemic *M. arboricola*, clarified its phylogenetic position among the sampled Sonerileae taxa, and enriched chloroplast genomic resources for *Medinilla*.

## Supplementary Material

Supplementary pictures and table.docx

## Data Availability

The genome sequence data that support the findings of this study are openly available in GenBank of NCBI (https://www.ncbi.nlm.nih.gov/) under the accession number PZ746659 (*M. arboricola*). The associated BioProject, SRA, and BioSample numbers are PRJNA1501936, SRP721295, and SAMN61961213, respectively.
