# Supplementary material for: The complete chloroplast genome of medinilla arboricola (melastomataceae) and its phylogenetic analysis
Source: Mitochondrial DNA B Resour. 2026 Oct 20;11(11):1354–8. doi: 10.1080/23802359.2026.2749904 (PMC13689795; doi:10.1080/23802359.2026.2749904)
Supplement: Supplementary file 1 [file TMDN_A_2749904_SM8404.docx]

Supplementary Figure S1. The quadripartite structure and coverage depth in Bandage.

1) Total genome length = 155517
2) Average depth = 1219.84×
3) Maximal depth = 1934×
4) Minimal depth = 9×

Supplementary Figure S2. The coverage plot across the assembled chloroplast genome.

Supplementary Figure S3. Schematic map of the cis-splicing genes.

Supplementary Figure S4. Schematic map of the trans-splicing genes.

Supplementary Table S1. The total, LSC, SSC, and IR region length of chloroplast genomes used

in this study.

| **Species** | **Accession**  **Number** | **Total length**  **(bp)** | **LSC (bp)** | **SSC (bp)** | **IR (bp)** |
| --- | --- | --- | --- | --- | --- |
| *Medinilla arboricola* | PZ746659 | 155,517 | 85,379 | 16,568 | 26,785 |
| *Blastus eglandulosus* | NC_068154 | 155,928 | 85,852 | 16,474 | 26,801 |
| *Blastus cochinchinensis* | MK814186 | 155,073 | 85.924 | 15,515 | 26,820 |
| *Phyllagathis erecta* | MK994811 | 156,339 | 85,814 | 16,947 | 26,789 |
| *Phyllagathis velutina* | MK994812 | 156,001 | 85,607 | 16,880 | 26,757 |
| *Medinilla fengii* | MK994809 | 155,841 | 85,629 | 16,632 | 26,790 |
| *Medinilla petelotii* | MK994847 | 155,673 | 85,355 | 16,712 | 26,803 |
| *Medinilla amplectens* | MK994882 | 155,529 | 85,372 | 16,524 | 26,813 |
| *Medinilla beamanii* | MK994880 | 155,271 | 85,025 | 16,633 | 26,803 |
| *Medinilla speciosa* | NC_068172 | 155,103 | 84,787 | 16,752 | 26,782 |
| *Medinilla tapete-magicum* | NC_081464 | 155,602 | 85,331 | 16,531 | 26,870 |
| *Pseudodissochaeta lanceata* | MK994850 | 156,790 | 86,448 | 16,860 | 26,741 |
| *Dissochaeta vacillans* | MK994856 | 156,200 | 85,844 | 16,871 | 26,743 |
| *Dissochaeta gracilis* | MK994855 | 156,227 | 85,866 | 16,879 | 26,741 |
